# Supplementary material for: A somatic genetic clock for clonal species
Source: Nat Ecol Evol. 2024 Jun 10;8(7):1327–36. doi: 10.1038/s41559-024-02439-z (PMC11239492; doi:10.1038/s41559-024-02439-z)
Supplement: Supplementary file 2 — Reporting Summary [file 41559_2024_2439_MOESM2_ESM.pdf]

nature portfolio

Corresponding author(s): Thorsten B.H. Reusch

Last updated by author(s): 2024/05/27

Reporting Summary

Nature Portfolio wishes to improve the reproducibility of the work that we publish. This form provides structure for consistency and transparency in reporting. For further information on Nature Portfolio policies, see our Editorial Policies and the Editorial Policy Checklist.

Statistics

For all statistical analyses, confirm that the following items are present in the figure legend, table legend, main text, or Methods section.

n/a	Confirmed	
☐	☒	The exact sample size (*n*) for each experimental group/condition, given as a discrete number and unit of measurement
☒	☐	A statement on whether measurements were taken from distinct samples or whether the same sample was measured repeatedly
☒	☐	The statistical test(s) used AND whether they are one- or two-sided<br>*Only common tests should be described solely by name; describe more complex techniques in the Methods section.*
☒	☐	A description of all covariates tested
☒	☐	A description of any assumptions or corrections, such as tests of normality and adjustment for multiple comparisons
☐	☒	A full description of the statistical parameters including central tendency (e.g. means) or other basic estimates (e.g. regression coefficient) AND variation (e.g. standard deviation) or associated estimates of uncertainty (e.g. confidence intervals)
☐	☒	For null hypothesis testing, the test statistic (e.g. *F*, *t*, *r*) with confidence intervals, effect sizes, degrees of freedom and *P* value noted<br>*Give P values as exact values whenever suitable.*
☒	☐	For Bayesian analysis, information on the choice of priors and Markov chain Monte Carlo settings
☒	☐	For hierarchical and complex designs, identification of the appropriate level for tests and full reporting of outcomes
☒	☐	Estimates of effect sizes (e.g. Cohen's *d*, Pearson's *r*), indicating how they were calculated

Our web collection on statistics for biologists contains articles on many of the points above.

Software and code

Policy information about availability of computer code

Data collection	Reference genome<br>- Zostera marina v3.1 NCBI BioProject PRJNA701932<br><br>Downloading SRA data:<br>fasterq-dump (sratoolkit.2.10.8-centos_linux64)<br><br>novel sequencing data:<br>Illumina HiSeq4000 and NovaSeq6000 genetic analyzers and proprietary data collection software
Data analysis	Modeling and simulations<br>- package for running clonal organism simulations at https://github.com/jessierenton/SomaticEvolution.jl<br>- custom-made scripts and simulation data, including the implemented Gillespie-algorithm, at https://github.com/jessierenton/somatic-genetic-clock<br><br>Analyzing histological images via confocal microscopy of the shoot apical meristem<br>- converting original confocal z-stack images (LIF) to TIFF files using open source (Fiji, https://fiji.sc).<br>- processing images, open source MorphoGraphX (MGX) v.2.0.1 (https://morphographx.org/software/)<br><br>Quality check of the raw Next-Generation Sequencing data<br>- FastQC v0.11.7 (https://www.bioinformatics.babraham.ac.uk/projects/fastqc/).

Filtering of the raw data
- BBDuk (https://jgi.doe.gov/data-and-tools/bbtools/bb-tools-user-guide/bbduk-guide/).

Mapping of short reads against reference genome
- sequence reads were mapped against the chromosome-level reference genome of Zostera marina V3.1 using BWA MEM (Burrows-Wheeler Alignment Tool v0.1.17).
- alignments were converted to BAM format and sorted using Samtools v1.11
- MarkDuplicates module in GATK4 v4.1.1.0 was used to remove duplicated reads (repository for GATK4 package at https://github.com/broadinstitute/gatk)
- filtering of the bam files using Samtools v1.11

Joint-calling of single nucleotide polymorphism for the Estonian clones
- HaplotypeCaller (GATK4 v4.1.1.0) was used to generate a GVCF format file for each sample, GVCF files were combined by CombineGVCFs (GATK4 v4.1.1.0).
- GenotypeGVCFs (GATK4 v4.1.1.0) was used to call genetic variants.
- VariantsToTable (GATK4 v4.1.1.0) was used to extract INFO annotations.
- quality filtering: marking by VariantFiltration (GATK4 v4.1.1.0) accd. to the criteria MQ < 40.0; FS > 60.0; QD < 10.0; MQRandSum > 2.5 or MQRandSum < -2.5; ReadPosRandSum < -2.5; ReadPosRandSum > 2.5; SOR > 3.0; DP > 1380.04 (2 * average DP), and those SNPs were excluded by SelectVariants (GATK4 v4.1.1.0).
- clone assignment (i.e. clonemates) based on shared heterozygosity (custom-made script at https://github.com/leiyu37/Detecting-clonemates.git).

Calling of somatic genetic variation (SNPs - single nucleotide polymorphisms)
- Mutect2 (GATK4 v4.1.1.0)
- Strelka2 (strelka-2.9.2.centos6_x86_64)

Mutational Spectra analysis
-germline: population-wise SNPs were extracted from 11705 core SNPs from Yu et al. Nature Plants 2023
--somatic SNPs were extracted from the 4 oldest genets detected in this data set
-mutational spectra were computed using the R-package Mutational.Patterns (no version, accessed Jan 2024)

Calculating the variable VRF50(X1, X2) as proxy for fixed somatic genetic variation
- custom-made scripts at https://github.com/leiyu37/SomaticGeneticClock.git

creating maps (Fig.4; Supplementary Fig. 15):
https://www.qgis.org/en/site/

For manuscripts utilizing custom algorithms or software that are central to the research but not yet described in published literature, software must be made available to editors and reviewers. We strongly encourage code deposition in a community repository (e.g. GitHub). See the Nature Portfolio guidelines for submitting code & software for further information.

Data

Policy information about availability of data

All manuscripts must include a data availability statement. This statement should provide the following information, where applicable:

- Accession codes, unique identifiers, or web links for publicly available datasets
- A description of any restrictions on data availability
- For clinical datasets or third party data, please ensure that the statement adheres to our policy

Custom-made scripts can be found at:
https://github.com/jessierenton/somatic-genetic-clock (simulation data)
https://github.com/jessierenton/SomaticEvolution.jl (analytical & population genetic calculations)
https://github.com/leiyu37/SomaticGeneticClock.git (bioinformatics)

Estonian eelgrass (Zostera marina) genets (=clones) (field sites KYD, SOE, KOI): BioProject no. PRJNA1025927
SRR26321797-SRR26321800
SRR26321805-SRR26321810

4-yr calibration clones: BioProject no. PRJNA1025927
SRR26321801-SRR26321804, SRR26321811, SRR26321812

17-yr calibration clones from California, Bodega Bay:
BioProject no. PRJNA806459
SRA accession nos. SRR18000159–SRR18000170.

Finnish clone (Ängsö):
BioProject no. PRJNA557092
SRA accession nos. SRR9879327- SRR9879353.

Eelgrass (Zostera marina) genets (=clones) sampled in global population genomics dataset (overview also given in Supplementary Data 1):
Bodega Bay, BB04, SRP193551
Bodega Bay, BB05, SRP193555
Bodega Bay, BB09, SRP193562
Bodega Bay, BB10, SRP193563

Japan South, JS03, SRP194687
Japan South, JS04, SRP193493
Northern Norway, NN02, SRP193666
Northern Norway,NN06, SRP193673
Northern Norway,NN07, SRP193674
Northern Norway,NN09, SRP194699
Northern Norway,NN10, SRP193677
Northern Norway,NN05, SRP193672
Northern Norway,NN08, SRP193675
Portugal, PO02, SRP193709
Portugal, PO05, SRP193715
Portugal, PO07, SRP194708
Portugal, PO08, SRP194712
Portugal, PO10, SRP194711
Portugal, PO11, SRP194715
Portugal, PO12, SRP194717
Portugal, PO03, SRP193716
Portugal, PO04, SRP193714
Portugal, PO06, SRP194707
Portugal, PO09, SRP194713
San Diego, SD04, SRP194696
San Diego, SD11, SRP193569
San Diego, SD06, SRP227665
San Diego, SD09, SRP193567
Washington State, WN04, SRP193698
Washington State, WN09, SRP227669
Washington State, WN06, SRP193703
Washington State, WN10, SRP227670

Human research participants

Policy information about studies involving human research participants and Sex and Gender in Research.

Reporting on sex and gender: na

Population characteristics: na

Recruitment: na

Ethics oversight: na

Note that full information on the approval of the study protocol must also be provided in the manuscript.

Field-specific reporting

Please select the one below that is the best fit for your research. If you are not sure, read the appropriate sections before making your selection.

☐ Life sciences ☐ Behavioural & social sciences ☒ Ecological, evolutionary & environmental sciences

For a reference copy of the document with all sections, see nature.com/documents/nr-reporting-summary-flat.pdf

Ecological, evolutionary & environmental sciences study design

All studies must disclose on these points even when the disclosure is negative.

Study description: The study combines agent based modeling on a hypothetical, generic clonal species, and empirical data of mixed origin on eelgrass (Zsotera marina) genets. In addition, confocal microscopy provided evidence for key parameters of growing eelgrass genets such as the stem cell population size, the founder population size and the ratio of symmetric vs. asymmetric cell divisions.
Data origin for empirical eelgrass data: Dataset of the 17-yr-old clones and of a global population genomic colelction of sites were from previous studies in which clone mates (i.e. ramets of the same genet), however, have not been analyzed . The dataset of the 4-yr-old calibration genets and the dataset of the Estonian clones were newly sequenced in this study.

Research sample: A research sample is a leaf shoot (or ramet) of the seagrass Zostera marina (=eelgrass).

Sampling strategy: Empirical data only: Samples were collected by snorkling or diving. Four-yr-old and 17-yr-old samples were collected from genets originally sampled at nearby locations (Kiel Bight, Germany, and Bodega Bay, California, USA; respectively), and cultured in the lab in large tanks (>500L) under flow through of ambient seawater, experiencing outside light conditions, and rooted in ambient sediment.

Data collection	Collectors are mentioned in the section on sampling permits. Sample extraction was performed at GEOMAR Kiel (Diana Gill and Lei Yu). DNA samples were sent to BGI Genomics (Hong Kong) for Illumina sequencing.
Timing and spatial scale	Sampling for the previous population genomics project was conducted between May 2016 and August 2017. Estonian samples were collected in August 2021. At a given site, a population was defined as continuous eelgrass meadow of at least 50 m across (parallel to shore). Samples for the 4-yr-old clones were collected from the lab In 2022.
Data exclusions	SNPs not passing the filtering criteria were excluded.
Reproducibility	SNP calling: two independent SNP calling approches were used (STRELKA2, Mutect2)<br>calibration of the somatic genetic clock: three (4-yr) and two (17-yr) old cultivated eelgrass genets (=clones) were used to obtain a calibration curve to age eelgrass genets (=clones) at other sites<br><br>identification of key covariates: an agent based model was used to examine the effects of branching rate (thus asexual generation time), number of founder cells, stem cell population size and the ratio asymmetric vs symmetric cell division. Within the parameter space of the study species eelgrass, a significant deviation of the somatic genetic clock from linearity is unlikely
Randomization	no randomization was required as the study question addressed identical clone mates
Blinding	no blinding was required

Did the study involve field work? ☒ Yes ☐ No

Field work, collection and transport

Field conditions	As our study builds upon genome polymorphism and differentiation that was emerging over hundreds to thousands of years, no environmental data were collected at the time of sampling
Location	All 20 sampling locations were geo-referenced, coordinates are listed in Supplementary Data 1 and below
Access & import/export	For all sites, sampling permits have been obtained by the relevant national or regional authorities where required. An e-mail string can be provided upon request between the local collaborators and the respective national authorities (NFP) with respect to an obligation or waiver of CBD or general sampling permit.<br><br>Populations with presence of genets = clones with >= 2 rsmets sampled:<br>- Japan South /J S /Pos 34.298N 132.916E. Sampling: collecting permit to Dr. Masakazu Hori, CBD-"Nagoya": see above<br>-Bodega Bay, USA / BB /Pos 38.320N 123.055W. Sampling: permit to Dr. John S Stachowicz through Dept Fish Wildlife CA. CBD-"Nagoya": non-signatory<br>-San Diego Bay, USA / SD / Pos 32.714N 117.225W. Sampling: permit to Dr. Kevin A Hovel through Dept Fish Wildlife CA. CBD-"Nagoya": non-signatory<br>-Røvika, Northern Norway / NN / Pos 67.268N 15.257E. Sampling: no permit required. CBD-"Nagoya": waiver<br>-Port Dinllaen, Wales, UK / WN/ 52.991N 4.450W. Sampling: waiver to Dr. Richard Unsworth by authorities as amount negligible. CBD-"Nagoya": waiver /collection before 1 July 2017<br>-Ria Formosa, Portugal /PO / 37.040N 7.910W saapling: no collection permit required. CBD-"Nagoya": collection before 1 July 2017<br>- Baltic Sea /Estonia, site Kuedema/KYD/pos 58.5331N 22.2380E: sampling permit through Prof. Jonne Kotta, Univ Tartu, CBD-"Nagoya": waiver<br>- Baltic Sea /Estonia, site Soela Strait /SOE/ Pos 58.6420N 22.6036E: sampling permit through Prof. Jonne Kotta, Univ Tartu, CBD-"Nagoya": waiver<br>- Baltic Sea /Estonia, site Koinastu /KOI /Pos 58.6184N 22.9928E: sampling permit through Prof. Jonne Kotta, Univ Tartu, CBD-"Nagoya": waiver<br><br>Populations without clones (not further analyzed in this ms):<br>Specific information is listed below for each site, from West to East:<br>- Japan North / JN /Pos 43.021N 144.903E. Sampling: collecting permit to Dr. Massa Nakaoka (in Japanese). CBD-"Nagoya": collection in August 2017 before implementation of CBD access regulation in Japan<br>- Alaska Safety Lagoon, USA /ASL / Pos 64.485N 164.762W. Sampling: no collecting permit required, waiver by U. S. Fish and Wildlife Service to Dr. David Ward & Dr. Sandra Talbot, CBD: non-signatory<br>-Alaska- Izembek Lagoon, USA /ALI / Pos 55.329N 162.821W. Sampling: no collecting permit required, waiver by U. S. Fish and Wildlife Service to Dr. David Ward & Dr. Sandra Talbot. CBD-"Nagoya": non-signatory<br>-Willapa Bay, Washington State, USA / WAS / Pos 46.474N 124.028W. Sampling: permit to Dr. Jennifer Ruesink through Wash Dept Natural Res. CBD-"Nagoya": non-signatory<br>-Quebec, Canada / QU / Pos 49.112N 68.176W. Sampling: permit to Dr. Mathieu Cusson through Fisheries and Oceans Canada. CBD-"Nagoya": non-signatory<br>-Massachusetts, USA / MA/ Pos 42.420N 70.915W. Sampling: permit to Dr. Randall Hughes through Massachusetts Division of Marine Fisheries. CBD-"Nagoya": non-signatory<br>-North Carolina, USA / NC / Pos 34.692N 76.623W. Sampling: permit to Dr. Joel Fodrie through North Carolina Division of Marine Fisheries. CBD-"Nagoya": non-signatory<br>-Torserød, West Coast of Sweden / SW / 58.313N 11.549E. Sampling: no permit required, waiver by Administrative County Board of Västra Götalands to Dr. Per-Olav Moksnes. CBD-"Nagoya": waiver<br>-Thau Lagoon, France / FR/ 43.447N 3.662E sampling: no collection permit required, waiver to Dr. Francesca Rossi. CBD-"Nagoya":

	waiver /collection before 1 July 2017<br>-Adriatic Sea, Croatia /CZ /Pos 44.212N 15.491E. sampling: no collection permit required, waiver to Dr. Stewart Schulz & Dr. Claudia Kruschel. CBD-"Nagoya": non-signatory
Disturbance	At each site, in an area of several 1000 m2, some leaf shoots of eelgrass were collected, representing <0.001% of all plants of the respective meadow. This level of disturbance is negligible compared to, for example, natural physical disturbance by storms or herbivory

Reporting for specific materials, systems and methods

We require information from authors about some types of materials, experimental systems and methods used in many studies. Here, indicate whether each material, system or method listed is relevant to your study. If you are not sure if a list item applies to your research, read the appropriate section before selecting a response.

Materials & experimental systems

n/a	Involved in the study
☒	☐ Antibodies
☒	☐ Eukaryotic cell lines
☒	☐ Palaeontology and archaeology
☒	☐ Animals and other organisms
☒	☐ Clinical data
☒	☐ Dual use research of concern

Methods

n/a	Involved in the study
☒	☐ ChIP-seq
☒	☐ Flow cytometry
☒	☐ MRI-based neuroimaging
